# Hallmark trials in ANCA-associated vasculitis (AAV) for the pediatric rheumatologist

**DOI:** 10.1186/s12969-019-0343-4

**Published:** 2019-06-26

**Authors:** Jennifer J. Y. Lee, Alhanouf Alsaleem, Grace P. K. Chiang, Elizaveta Limenis, Watchareewan Sontichai, Rae S. M. Yeung, Jonathan Akikusa, Ronald M. Laxer

**Affiliations:** 1 Department of Pediatrics, Division of Rheumatology, Hospital for Sick Children and University of Toronto, 555 University Avenue, Toronto, ON M5G 1X8 Canada; 2 Department of Pediatrics and Adolescent Medicine, Alice Ho Miu Ling Nethersole Hospital, HKSAR, Tai Po, Hong Kong; 3 Department of Pediatrics, Faculty of Medicine, Chiang Mai University, Chiang Mai, Thailand; 4 Department of Rheumatology, The Royal Children’s Hospital, Melbourne, Australia

**Keywords:** Pediatric, Vasculitis, Anti-neutrophil cytoplasmic antibody-associated Vasculitis, Management

## Abstract

Anti-neutrophil cytoplasmic antibody (ANCA)-associated vasculitis (AAV) refers to a complex group of systemic vasculitides that are characterized by primary small-to-medium sized blood vessel inflammation with the presence of autoantibodies known as ANCA. AAV diseases include Granulomatosis with Polyangiitis (GPA), Eosinophilic Granulomatosis with Polyangiitis (EGPA), and Microscopic Polyangiitis (MPA). AAVs are challenging conditions associated with high cumulative disease and treatment related morbidity and mortality. Given its rarity and the resulting paucity of pediatric-specific clinical trial evidence, pediatric rheumatologists have had to often extrapolate from adult literature for management and therapeutic decisions. The aim of this review is to provide a comprehensive overview of the important findings and overall conclusions of critical landmark clinical trials in the induction and maintenance treatments in adult AAV for the pediatric rheumatologist. This review also highlights the outcomes of recent pediatric AAV observational studies and discusses the future research priorities in pediatric AAV management.

## Background

Systemic vasculitis is a challenging and complex multi-organ disease that results in primary inflammation of the blood vessel wall. Anti-neutrophil cytoplasmic antibody (ANCA)-associated vasculitis (AAV) is a group of systemic vasculitides that is characterized by small-to-medium sized blood vessel inflammation with the presence of autoantibodies known as ANCA. AAV diseases include Granulomatosis with Polyangiitis (GPA), Eosinophilic Granulomatosis with Polyangiitis (EGPA), and Microscopic Polyangiitis (MPA). AAVs are one of the more common types of systemic vasculitis encountered by pediatric rheumatologists. Proper treatment of this condition is critical as the mortality of untreated AAV can be up to 80% [1, 2]. Given the paucity of clinical trials in pediatric AAV, pediatric rheumatologists have relied on adult AAV evidence for management. In this review, we highlight key findings of critical landmark trials in AAV for the pediatric rheumatologist.

## Disease activity assessments

Standardized tools are important in measuring disease activity and damage; they also help guide treatment decisions in rheumatic diseases. Numerous instruments have been developed to measure disease activity in AAV [3–5]. These measurements are often used to define primary or secondary outcomes in AAV trials. Thus, it is important for pediatric rheumatologists to familiarize themselves with these tools.

### Birmingham Vasculitis activity score

The Birmingham Vasculitis Activity Score (BVAS), originally published in 1994 and then revised in 1997 and 2009, is the most widely used tool in clinical practice and trials [6, 7]. The BVAS is a composite score that evaluates 56 clinical features from 9 organ systems that are attributed to active vasculitis. Each item is weighted according to the severity. A score of 0 is often adopted as the definition of disease remission in studies. The revised BVAS acknowledges persistent symptoms in addition to new and worsening symptoms. A variation of the BVAS available for GPA patients is known as the BVAS/WG [8]. This score has greater disease specificity in patients with GPA but cannot be generalized to other types of systemic vasculitis.

### Pediatric Vasculitis activity score

There had been no validated tool for assessment of disease activity in pediatric patients with systemic vasculitis until 2012 [9], when international collaborative efforts led to the development and validation of a pediatric vasculitis assessment tool. The Pediatric Vasculitis Activity Score (PVAS) is modified from the BVAS. In the PVAS, 22 original BVAS items were redefined and 8 new items were added, resulting in 64 clinical items grouped under 9 organ systems. Every item has an assigned score in the ‘new/worse’ and ‘persistent’ scale. This score was used in a recent study to measure the early outcomes in children with AAV [10].

## Disease damage assessments

Measurement of damage is an essential component in the follow-up assessments of chronic disease. The Vasculitis Damage Index (VDI) [11] is an unweighted scoring system comprising 64 items grouped under 11 organ-based systems. Damage is defined as an irreversible change lasting for more than 3 months. The VDI is a cumulative index and can only remain static or increase over time. The damage recorded needs to occur after the vasculitis diagnosis, but do not need to be attributable to the diagnosis (e.g. might be related to treatment).

There is no validated tool to assess disease damage in children with vasculitis. However, the Pediatric Rheumatology European Society (PRES) Vasculitis Working Group and Childhood Arthritis & Rheumatology Research Alliance (CARRA) are working toward validating a formal pediatric modification of the VDI [10] – PVDI, which has been piloted in some studies.

## Treatment overview

Treatment of AAV is generally categorized into two phases: induction and maintenance. Induction therapy refers to the therapies required to achieve disease remission. After achieving remission, maintenance therapy is initiated to prevent relapses. This review discusses induction therapy for severe and limited AAV separately, followed by maintenance therapy trials.

The European League Against Rheumatism (EULAR) has previously described more specific disease severity definitions by expert consensus in order to conduct trials with more homogeneous AAV patient populations, and to make trials comparable **(**Table 1**)** [12]**.** For clinically relevant purposes, severe (also described as generalized) AAV is defined as presence of life- or major organ-threatening manifestations. The Canadian Vasculitis Research Network (Canvasc) recommendations on AAV management provides clinical examples describing major organ-threatening manifestations, which may include severe and progressive kidney involvement, alveolar haemorrhage resulting in severe hemoptysis, severe gastrointestinal (e.g. intestinal bleeding), cardiac (e.g. heart failure due to pericarditis or myocarditis), central nervous system (e.g. rapidly progressive neuropathy), or ocular involvement (e.g. orbital pseudotumor) [13]. Limited AAV is often defined as localized involvement without organ-threatening manifestations. Patients with constitutional symptoms are generally included. Patients with renal or pulmonary manifestations may be included with the caveat that the organ manifestations do not result in threatened organ function (e.g. pulmonary infiltrates without severe hemoptysis or mildly reduced kidney function with a Cr < 120 without evidence of casts or significant proteinuria).

**Table 1:** Disease Severity Definitions

Study Group	Clinical Subgroup	Systemic Vasculitis Outside Ears, Nose, Throat and Lungs	Threatened Vital Organ Function	Other Definitions	Serum Creatinine (umol/L)
EUVAS	Localized	No	No	No constitutional symptoms, ANCA typically negative	< 120
	Early Systemic	Yes	No	Constitutional symptoms present, ANCA-positive or negative	< 120
	Generalized	Yes	Yes	ANCA-positive	< 500
	Severe	Yes	Organ Failure	ANCA-positive	> 500
	Refractory	Yes	Yes	Refractory to standard therapy	Any
WGET Research Group	Limited	Allowed, but not required	No	Not severe	< 124, if hematuria, but no red blood cell casts present
	Severe	Yes	Yes	Organ- or life-threatening disease	Any

From Hellmich et al., EULAR Recommendations for conducting clinical studies and/or clinical trials in systemic vasculitis: focus on anti-neutrophil cytoplasm antibody-associated vasculitis. [12]

## Induction trials

### Induction trials for severe disease

Several trials have addressed induction regimens for severe AAV (Table 2).

**Table 2:** Induction Trials in AAV

Reference, Country	Study Design	Patient Selection	Experiment	Comparators	Primary Outcome	Results	Adverse Events
NORAM, Groot et al., 2005, Germany	Unblinded, prospective RCT	GPA or MPA limited/non-severe disease	MTX PO 15 mg/week escalated to a maximum of 20–25 mg/week by 12 weeks, until month 10, then tapered and discontinued by month 12 Prednisone 1 mg/kg/day, tapered to 7.5 mg by 6 months, discontinued by 12 months	CYC PO 2 mg/kg/day (maximum 150 mg/day) × 3–6 months until remission then 1.5 mg/kg to month 10, then tapered and discontinued by month 12 Prednisone 1 mg/kg/day, tapered to 7.5 mg by 6 months, discontinued by 12 months	Remission within 6 months	MTX (89.8%) CYC (93.5%)	83 patients: adverse events 68 patients: mild/moderate infection 15 patients: severe infection MTX: liver toxicity (p 0.036) CYC: leukopenia (p 0.012)
MEPEX, Jayne et al., 2007, Europe	RCT	GPA, MPA with severe renal vasculitis	PLEX 60 ml/kg for 7 cycles within 14 days CYC PO 2.5 mg/kg/day, reduced to 1.5 mg/kg/day at 3 months and discontinued at 6 months Prednisone 1 mg/kg/day tapered until 10 mg/day from 5 to 12 months	Pulse GC 1 g for 3 days CYC PO 2.5 mg/kg/day, reduced to 1.5 mg/kg/day at 3 months and discontinued at 6 months Prednisone 1 mg/kg/day tapered until 10 mg/day from 5 to 12 months	Renal recovery at 3 months	PLEX 69% IV GC 49%	No difference between 2 groups PLEX: 50% Pulse GC: 48%
CYCLOPS, Groot et al., 2009, Europe	Open label RCT	GPA, MPA, renal limited MPA (GFR < 500)	CYC IV pulses 15 mg/kg, given 2 weeks apart, followed by pulses at 3-week interval until remission, and then for 3 months Prednisone 1 mg/kg/day tapered to 12.5 mg by 3 months then 5 mg at 18 months	CYC PO 2 mg/kg/day until remission, followed by 1.5 mg/kg/day for 3 months Prednisone 1 mg/kg/day tapered to 12.5 mg by 3 months then 5 mg at 18 months	Time to remission	87.9% achieved remission by 9 months (no difference between the two groups, 88% in the IV group, 87.7% in the PO group) Relapses: CYC IV: 13 patients CYC PO: 6 patients CYC IV: lower cumulative dose (p 0.001)	IV group: less leukopenia (26% vs 45%) Death: CYC IV: 5 patients CYC PO: 9 patients No difference in the rate of life threatening events
RITUXVAS, Jones et al., 2010, Europe/Australia	Open label RCT	Newly diagnosed AAV with evidence of renal involvement	RTX, 375 mg/m^2^ weekly for 4 weeks plus CYC IV 15 mg/kg with 1st and 3rd dose Pulse GC 1 g, followed by prednisone 1 mg/kg/day, tapered to 5 mg by 6 months	CYC IV 15 mg/kg every 2 weeks for the first 3 doses then every 3 weeks, until remission (3–6 months) then AZA 2 mg/kg to end of study (12 months) prednisone 1 mg/kg/day, tapered to 5 mg by 6 months	Sustained remission at 12 months Time to remission	RTX was not superior to CYC. Sustained remission: RTX: 76% CYC: 82% Median time of remission: RTX: 90 days CYC: 94 days	Similar rate of adverse events RTX: 42% CYC: 36% Similar death rate in both groups: 18%
RAVE, Stone et al., 2010, USA	Double blinded RCT	Severe AAV (period of 6 months)	RTX 375 mg/m^2^ weekly for 4 weeks then placebo AZA for 18 months Pulse GC 1 g for 1–3 doses followed by prednisone 1 mg/kg/day, discontinued by 5 months	CYC PO 2 mg/kg/day until remission (3–6 months) then AZA for 18 months Pulse GC 1 g for 1–3 doses followed by prednisone 1 mg/kg/day, discontinued by 5 months	Disease remission off steroids by 5 months	RTX was not inferior to CYC. RTX regimen was superior to CYC in inducing remission in previously relapsing disease	No difference in the number of adverse events CYC higher rate for leukopenia (10% vs 3%)
MYCYC, Jones et al., 2019, UK	Open label RCT	Newly diagnosed AAV, non- life threatening	MMF 2–3 g (BSA dose for patients < 17 years old) Prednisone 1 mg/kg/day tapered to 5 mg by 6 months	CYC IV 15 mg/kg, given 2 weeks apart, followed by pulses at 3-week intervals until remission, and then for 3 months Prednisone 1 mg/kg/day tapered to 5 mg by 6 months	Remission by 6 months	MMF (67%) was not inferior to IV CYC (61%). Relapse rate higher in MMF (33%) vs IV CYC (19%)	No significant difference in serious adverse events between two groups MMF (50%) IVCYC (40%)

*RCT* Randomized Controlled Trial, *AAV* Anca associated vasculitis, *GPA* Granulomatosis with Polyangiitis, *MPA* Microscopic Polyangiitis, *PLEX* Plasma exchange, *MTX* Methotrexate, *CYC* Cyclophosphamide, *PO* Oral, *IV* Intravenous, *GC* Glucocorticoids, *GFR* Glomerular Filtration Rate, *RTX* Rituximab, *MMF* Mycophenolate mofetil

Cyclophosphamide (CYC) is one of the most commonly used and well-studied induction therapies. All CYC trials were conducted with one shared aim: to achieve disease remission and minimize medication toxicity. CYCLOPS was an open label multicenter randomized controlled trial (RCT) that evaluated the effect of intermittent intravenous (ivCYC) versus daily oral CYC (poCYC) [14]. The primary outcome was time to remission, defined as the absence of new or worse signs of disease activity on the BVAS and no more than 1 item indicating persistent disease activity (BVAS < 1). The time to remission did not differ between groups (median 3 months). The authors concluded that the use of ivCYC in comparison to poCYC had similar efficacy in achieving remission, but ivCYC therapy had the advantage of having a lower toxicity profile with a reduced cumulative dose. However, a long-term follow-up study with a median duration of 4.3 years showed higher relapse rates with ivCYC compared to poCYC. Interestingly, anti-proteinase-3 (PR3) positive patients had higher relapse rates in both groups.

Rituximab (RTX), an anti-CD20 monoclonal antibody and B-cell depleting agent, has emerged as a new therapeutic agent for AAV. RITUXVAS is a multicenter RCT in which newly diagnosed AAV patients with renal disease were randomized to receive ivCYC for 3–6 months, or RTX weekly for 4 weeks plus ivCYC with the first and third RTX infusion. All patients received 1 g of methylprednisolone followed by a tapering course of oral corticosteroids, with reduction to 5 mg by 6 months [15]. The primary outcome was rates of sustained remission (BVAS of 0 for at least 6 months) and rates of serious adverse events (AEs) at 12 months. The RTX-based induction regimen was not superior to ivCYC. The long-term follow-up study also reported no difference in disease remission, AEs, or mortality [16].

The RAVE (Rituximab in ANCA-Associated Vasculitis) trial evaluated RTX in comparison to poCYC utilizing a non-inferiority trial design [17]. This trial differed from the RITUXVAS trial in that the participants of the RAVE trial were younger with less severe renal disease. Furthermore, the comparator agent was oral as opposed to intravenous CYC. The ambitious primary outcome was to achieve disease remission (BVAS of 0) off corticosteroids by 6 months. Both groups had similar corticosteroid regimens. Sixty-four percent of patients in the RTX group had disease remission in comparison to 53% in the poCYC group. This result met the definition for non-inferiority. However, remission rates were overall lower in comparison to other studies, possibly attributable to the earlier discontinuation of corticosteroids. In subsequent analyses, subgroups of patients with either relapsing disease at baseline or those with anti-PR3 positivity achieved higher response rates with RTX when compared to CYC. A long-term follow-up of the RAVE cohort showed that in patients with severe AAV without organ failure, RTX regimen was equivalent to CYC in maintaining disease remission after 18 months [18].

Plasma exchange (PLEX) is a non-pharmacologic treatment considered in patients with severe AAV. The MEPEX trial compared the addition of either IV corticosteroids or PLEX in patients with AAV who had severe renal vasculitis (serum creatinine > 500 μmol/L or dialysis dependence). The primary outcome was renal recovery (dialysis independence) at 3 months, which was achieved in 49 and 69% in the corticosteroid and PLEX arm, respectively [19]. PLEX decreased the incidence of end stage renal disease (ESRD) from 43 to 24% at 12-months, but this difference was lost after long-term follow-up [20]. PEXIVAS is an international, open-label, two-by-two factorial design study that recruited patients with new or relapsing severe AAV to investigate the use of adjunctive PLEX with standard therapy (CYC or RTX) and two different corticosteroid regimens (standard or low dose) [19]. The primary outcome was a composite measure of death from any cause or ESRD. Preliminary results from 704 patients found that the primary outcome occurred in 28% in the PLEX arm compared to 31% in the no-PLEX arm. [21]. Interestingly, varying the steroid regimens did not result in a difference to the primary outcome.

### Induction trials for limited disease

There has been interest in identifying safer immunosuppressive regimens in the management of patients with limited AAV. The NORAM (Non-Renal vasculitis Alternatively treated with Methotrexate), a non-blinded RCT, hypothesized that induction with oral Methotrexate (poMTX) can spare the toxicity of poCYC in early systemic AAV [22]. The primary outcome was disease remission by 6 months, with tapering of induction therapy by 12 months. All patients were treated with the same corticosteroid regimen. The NORAM trial demonstrated that poMTX was not inferior to poCYC. However, at 18 months, relapse rates were significantly higher in the poMTX group, suggesting that when given as an alternative to poCYC, poMTX may need to be given for longer than 12 months. Long-term follow-up of the cohort (median 6 years) found no difference with respect to AEs between the two regimens and higher relapse rates in the poMTX-treated group [23].

Mycophenolate mofetil (MMF) has been studied for induction therapy for limited disease. MYCYC (MMF versus CYC for remission induction of AAV) is a randomized non-inferiority trial that compared MMF with ivCYC. The primary outcome was the proportion of patients achieving remission (BVAS of 0) by 6 months. The MMF group received doses ranging from 2 to 3 g and the ivCYC group was treated with a similar regimen used in the CYCLOPS trial. Both groups received the same corticosteroid regimen. Sixty-seven percent of patients in the MMF group achieved the primary outcome in comparison to 61% in the ivCYC group. Following remission, relapses occurred significantly more frequently with MMF (33%) compared to ivCYC (19%). The authors concluded that MMF was non-inferior to ivCYC, but that MMF may result in more relapses [24].

## Maintenance trials

Given the concerns of utilizing CYC long-term, such as increased risk of malignancy and infertility, investigators have evaluated the use of less toxic immunosuppressants as alternatives (Table 3). In the CYCAZAREM (CYC versus AZA for Early REMission phase of vasculitis) trial, patients with a newly diagnosed severe AAV and a serum creatinine of < 500 μmol/L in whom remission had been achieved within 3–6 months, were randomly assigned to continue poCYC or switch to azathioprine (AZA) for 12 months. All subjects were then switched to a lower dose of AZA, which continued to the end of the study (18 months). Both groups continued a tapering course of corticosteroids. The primary outcome was major and minor relapse rate at 18 months. Relapse rates were not significantly different between groups [25]. There was no difference in severe AEs, although the study was not powered to detect differences in AE rates. Long-term follow-up (median 8.5 years) revealed a trend for worse outcomes in the AZA group in terms of relapses and development of ESRD but these were not statistically significant [26].

**Table 3:** Maintenance Trials in AAV

Reference, Country	Study Design	Patient Selection	Induction	Experiment	Comparators	Primary Outcome	Results	Adverse Events
CYCAZAREM, Jayne et al., 2003, Europe	Open label RCT	Newly diagnosed GPA, MPA, renal-limited vasculitis (serum Cr < 500 μmol/L)	CYC PO and GC	AZA 2 mg/kg/day, tapered to 1.5 mg/kg/day at month 12, and discontinued at month 18 and Prednisolone 10 mg/day until month 12, tapered to 7.5 mg/day until month 18	CYC PO 1.5 mg/kg/day until month 12 then AZA 1.5 mg/kg/day until month 18 and Prednisolone 10 mg/day until month 12, tapered to 7.5 mg/day until month 18	Relapse rate (major and minor)	15.5% in AZA, 13.7% in CYC (p 0.65)	- Severe AEs: 11% in AZA, 10% in CYC (p 0.94)
Metzler et al., 2007, Germany	Open label RCT	Generalized GPA Serum Cr < 115 μmol/L	CYC PO and GC	LEF 100 mg for 3 days, followed by 20 mg/day until week 4 then 30 mg/day and Prednisolone 10 mg/day or below and tapered by 2.5 mg/month until 5 mg and by 1 mg/month thereafter	MTX PO 7.5 mg/week, gradually increased to 20 mg/week after week 8 and Prednisolone 10 mg/day or below and tapered by 2.5 mg/month until 5 mg and by 1 mg/month thereafter	Relapse rate (major and minor)	23% in LEF, 46% in MTX (p 0.09)	- No difference of AEs in LEF and MTX - 15% in LEF were withdrawn: hypertension, leukopenia, peripheral neuropathy
WEGENT, Pagnoux et al., 2008, France	Open label RCT	Newly diagnosed GPA or MPA with systemic involvement	CYC IV and GC	AZA 2 mg/kg/day for 12 months then withdraw over 3 months and Prednisolone 12.5 mg at 6 months and tapered to 5 mg/day at 18 months and discontinued at month 24	MTX 0.3 mg/kg, increased every week to 25 mg/week for 12 months then withdraw over 3 months and Prednisolone 12.5 mg at 6 months and tapered to 5 mg/day at 18 months and discontinued at month 24	AEs causing death or drug discontinuation	11% in AZA, 19% in MTX (p 0.21) Death: 1 in MTX (hematotoxicity and sepsis) Relapse: 36% in AZA vs 33% in MTX (p 0.71)	- Any AEs: 46% in AZA, 56% in MTX (p 0.29)
IMPROVE, Hiemstra et al., 2010, Europe	Open label RCT	Newly diagnosed GPA, MPA	CYC PO/IV and GC ± PLEX	MMF 2 g/day, reduced to 1.5 g/day after 12 months, 1 g/day after 18 months and discontinued after 42 months and Prednisolone until month 24	AZA 2 mg/kg/day, reduced to 1.5 mg/kg after 12 months, 1 mg/kg after 18 months, and discontinued after 42 months and Prednisolone until month 24	Relapse-free survival	55% in MMF, 37.5% in AZA (p 0.03, HR 1.69)	- Severe AEs: 7.5% in MMF, 16% in AZA (p 0.12) - Death: 1 in MMF (fungal septicemia), 1 in AZA (sudden cardiac death)
MAINRITSAN, Guillevin et al., 2014, France	Open label RCT	Newly diagnosed or relapsing GPA, MPA, renal-limited vasculitis	CYC IV and GC	RTX 500 mg at day 0, 14, month 6, 12, 18 Prednisone 5 mg/day for at least 18 months	AZA 2 mg/kg/day 12 months then 1.5 mg/kg 6 months, then 1 mg/kg 4 months Prednisone 5 mg/day for at least 18 months	Major relapse rate at 28 months	5% in RTX, 29% in AZA (p 0.002, HR 6.61) Minor relapse: 11% in RTX, 16% in AZA (p 0.43)	- Severe AEs: no significant difference - Death: 2 in AZA (sepsis and pancreatic cancer)
REMAIN, Karras et al., 2017, Europe	Open label RCT	GPA, MPA, renal-limited vasculitis	CYC and GC ± PLEX	Continuation group: AZA 1 mg/kg/day to end of study and prednisolone 5 mg/day for 12 months then tapered and discontinued by 24 months	Withdrawal group: AZA 0.75 mg/kg/day for 3 months and prednisolone 5 mg/day, tapered and discontinued by month 5	Relapse rate (major and minor) by 30 months	63% in withdrawal group, 22% in continuation group (*p* < 0.0001, RR 2.84) No difference of relapse severity	- Severe AEs: 15% in continuation group, 6% in withdrawal group (p 0.13)
MAINRITSAN2, Charles et al., 2018, France	Open label RCT	Newly diagnosed or relapsing GPA, MPA	GC and CYC, RTX or MTX	Individually tailored: RTX 500 mg at randomization and then reinfused based on CD19+ B lymphocytes and ANCA titers until month 18 and low-dose prednisone	Fixed-schedule: RTX 500 mg at day 0, 14, month 6, 12, 18 and low-dose prednisone	Relapse rate at month 28	17.3% in individually tailored group, 9.9% in fixed-schedule group (p 0.22)	- Severe AEs: 32.1% in individually tailored group, 38.3% in fixed-schedule group (p 0.51)
BREVAS, Jayne et al., 2019	Double blind RCT	Newly diagnosed or relapsing GPA, MPA with ANCA positivity	GC and either CYC or RTX	Belimumab IV 10 mg/kg and AZA 2 mg/kg/day and low-dose GC (≤10 mg/day)	Placebo and AZA 2 mg/kg/day and low-dose GC (≤10 mg/day)	Time to first protocol-specified event (BVAS ≥6, presence of ≥1 major BVAS, or receipt of prohibited medications for any reason)	18.9% in belimumab, 21.2% in control (p 0.89)	- Serious AEs: 34% in belimumab, 30.8% in placebo

*RCT* Randomized Controlled Trial, *GPA* Granulomatosis with Polyangiitis, *MPA* Microscopic Polyangiitis, *Cr* creatinine, *CYC* Cyclophosphamide, *PO* oral, *GC* Glucocorticoids, *AZA* Azathioprine, *AE* Adverse event, *LEF* Leflunomide, *MTX* Methotrexate, *IV* intravenous, *PLEX* Plasma exchange, *MMF* Mycophenolate mofetil, *HR* Hazard ratio, *RTX* Rituximab, *ANCA* Anti-neutrophil cytoplasmic antibody, *BVAS* Birmingham Vasculitis Activity Score

While the CYCAZAREM study validated AZA as a suitable alternative to poCYC for maintenance, the optimal duration of AZA treatment was not examined. The REMAIN (prolonged REmission-MAINtenance therapy in systemic vasculitis) trial concluded that prolonged maintenance therapy with AZA and low dose corticosteroids to 48 months from diagnosis resulted in a 3-fold reduction in the frequency of relapses compared with withdrawal of AZA and corticosteroids by 24 months [27]. Moreover, the continuation group had improved renal survival with reduced incidence of ESRD. In a RCT (AZA-ANCA trial) comparing standard and extended AZA maintenance therapy in patients with PR3-AAV, patients treated with longer treatment duration (4 years after diagnosis and tapered thereafter) had a lower relapse rate, albeit not significant, compared with those treated with standard treatment (1 year after diagnosis and tapered thereafter) [28]. However, this trial was terminated prematurely given slow patient recruitment and did not achieve an adequate sample size. Therefore, although not definitively proven, a longer duration of maintenance therapy may lead to better outcomes.

The use of MTX as an alternative maintenance agent with possibly less toxicity and equal or perhaps greater efficacy than AZA was examined in the WEGENT trial. In the WEGENT trial, AAV patients in remission were randomized to either AZA or MTX and a tapering oral steroid course [29]. The rate of AEs causing death or study withdrawal was the same between the two groups, indicating MTX was similar in toxicity with AZA. Relapse rates were also similar confirming MTX as a viable alternative to AZA. In the follow-up study, 10-year overall survival rates, total number of relapses, relapse rates and AEs did not differ significantly [30].

Other immunosuppressive agents, including MMF and leflunomide, have also been studied. The IMPROVE (International MMF Protocol to Reduce Outbreaks of Vasculitides) trial compared the efficacy of MMF versus AZA for maintenance of remission in AAV patients in whom remission had been induced with corticosteroids and CYC, with or without methylprednisolone pulses and PLEX. MMF was significantly less effective at preventing relapses when compared to AZA after a median follow-up of 39 months [31]. In a RCT comparing leflunomide and MTX, leflunomide was found to be more effective than MTX in preventing major relapses but was associated with more AEs [32].

The MAINRITSAN (Maintenance of Remission using Rituximab in Systemic ANCA-Associated Vasculitis) trial is the first RCT to evaluate RTX for maintenance therapy. After achieving remission, patients were randomly assigned to receive either RTX at 0 and 2 weeks following randomization then every 6 months until 18 months or AZA until 22 months. Both groups were treated with corticosteroids for at least 18 months. There were significantly fewer major relapses at 28 months in the RTX group compared with the AZA group (5% versus 29%) [33]. In the long-term study, RTX remained superior to AZA up to 60 months, with greater rates of relapse-free and overall survival [34]. The MAINRITSAN2 compared an individually tailored RTX regimen with fixed-schedule regimen [35]. Patients in the tailored-infusion arm received RTX at randomization and received repeat infusions based on lymphocyte counts and ANCA titers until 18 months. The fix-scheduled arm received the same regimen from the original MAINRITSAN trial. There was no significant difference between the number of relapses but the tailored-infusion arm received fewer number of infusions overall.

The BREVAS (Belimumab in Remission of Vasculitis) is a recent RCT that evaluated the efficacy of belimumab, a monoclonal antibody against B lymphocyte stimulator, as an adjunctive therapy to a regimen of AZA with low-dose corticosteroids [36]. Overall, the addition of belimumab did not reduce relapses.

### Eosinophilic granulomatosis with Polyangiitis (EGPA)

EGPA treatment recommendations are less robust due to the lack of RCTs. The treatment is often inferred from GPA/MPA trials, although EGPA patients were either excluded or present in small numbers in these studies.

Only a few trials have studied immunosuppressive therapy in larger numbers of EGPA patients **(**Table 4**)** [37–40]. The most commonly studied agent for EGPA has been CYC, although studies have largely looked at variations in CYC regimens as opposed to comparing the efficacy of CYC to other immunosuppressants. Currently, the EGPA Consensus task force recommends the use of CYC for patients with organ threatening disease for induction [41].

**Table 4:** EGPA Trials

Reference, Country	Study Design	Patient Selection	Experiment	Comparators	Primary Outcome	Results	Adverse Events
Induction Trials							
Guillevin et al., 1991, France	RCT	PAN or EGPA	*Group A:* GC (1 mg/kg/day for 1 month then tapered) and PLEX (12 exchanges in first 2 months) (*n* = 36)	*Group B:* GC only (1 mg/kg/day for 1 month then tapered) (*n* = 42)	Control of disease (Recovery, remission), or death	78 patients (18 EGPA patients) Primary Outcomes: *Complete remission:* 27 in group A, 29 in group B *Relapse:* 10/36 in group A, 8/42 in group B *Death:* 6 in group A, 9 in group B. No significant difference in 7 year cumulative survival rates	Deaths part of primary outcome
Guillevin et al., 1995, France	RCT	Severe PAN or EGPA with features of poor prognosis	*Additional use of PLEX:* GC (15 mg/kg/day IV for 3 days, then PO 1 mg/kg/day for 1 month with tapering) and IV CYC pulse (600 mg/m2 every month for a year) and PLEX (9 exchanges in 3 weeks) (*n* = 34)	GC (15 mg/kg/day IV for 3 days, then PO 1 mg/kg/day for 1 month with tapering) and IV CYC pulse (600 mg/m2 every month for a year) (*n* = 28)	Relapse or remission or death	62 patients (14 EGPA patients) Primary Outcomes: *Remission*: 16 in no PLEX group, 22 in PLEX group *Relapse*: 4 (14%) in no PLEX group, 3 (9%) in PLEX group *Death:* 7 (25%) in no PLEX group, 4 (11.8%) in PLEX group	AEs in PLEX group: pulmonary TB in 3 patients, pneumonia in 3 patients, acute sigmoiditis in 1 patient, and septicemia in 2 patients
Gayraud et al., 1997, France	RCT	Good prognosis PAN or EGPA	*Group A:* GC (1 mg/kg/day for 1 month, decreased by 2.5 mg every week until 10 mg/day. At 6 months, decreased by 1 mg/week) and oral CYC (2 mg/kg/day) for 12 months (*n* = 12)	*Group B:* GC (1 mg/kg/day for 1 month, decreased by 2.5 mg every week until 10 mg/day. At 6 months, decreased by 1 mg/week) and IV pulse CYC (600 mg/m2 every month for a year) (*n* = 13)	Complete remission rates	25 patients (8 EGPA patients) Primary Outcome: Complete remission in 9 (75%) in Group A, 10 (77%) in Group B Secondary Outcomes: *Relapses:* 2 in each group *Failures:* 1 in each group	10 patients in group A experienced AEs, 8 in group B experienced AEs
Cohen et al., 2007, France	RCT	EGPA patients with features of poor prognosis	*Shorter CYC regimen:* GC (15 mg/kg/day IV for 3 days, then 1 mg/kg/day oral for 1 month followed by tapering) and 6 IV CYC pulses (600 mg/m2 every 2–4 weeks) (*n* = 23)	GC (15 mg/kg/day IV for 3 days, then 1 mg/kg/day oral for 1 month followed by tapering) and 12 IV CYC pulses (600 mg/m2 every 2–4 weeks) (*n* = 25)	Complete remission rates	48 patients Primary Outcome: Complete Remission in 21 (91%) in 6 pulse group vs 21 (84%) in 12 pulse group (NS) Secondary Outcomes: *Relapses:*18 (86%) ha a relapse in the 6 pulse group, 13 (62%) had a relapse in the 12 pulse group *Deaths:* 2 patients died in each group	Similar AE between groups (13 patients in the 12-pulse group compared to 11 patients in the 6-pulse group)
Ribi et al., 2008, France	RCT	EGPA patients without features of poor prognosis who had treatment failure or relapse on GC alone (could not taper below 20 mg or those who experienced relapse)	AZA (2 mg/kg/day) for 6 months (*n* = 8)	6 IV CYC pulses (600 mg/m2 every 2–4 weeks) (*n* = 10)	Complete remission rates	72 patients, but 19 patients met inclusion criteria Primary Outcome: 5 (50%) in the CYC group experienced remission, 7 (78%) in the AZA group achieved remission (NS)	CYC AE: Hemorrhagic cystitis (n = 1), Infertility (n = 1) AZA AE: Skin rash (n = 2), elevated LFTs (n = 1)
Pagnoux et al., 2015, France	Nonblinded, RCT	≥ 65 years old with new diagnosis of PAN, GPA, MPA, or EGPA	*Shorter GC duration and lower CYC doses:* GC for 9 months (started at 1 mg/kg/day and tapered) and six 500 mg IV CYC pulses every 2–3 weeks	26 months of GC (3 additional pulses for consolidation before maintenance for at least 18 months) and 500 mg/m2 IV CYC pulses every 2–3 weeks	Occurrence of ≥ 1 SAE, including deaths from all causes, during the 3 years of follow-up	104 patients (14 EGPA patients). Primary Outcome: 32 (60%) of the patients in the experimental arm had ≥ 1 SAE versus 40 (78%) of the patients in the conventional arm (*p* = 0.04). 9 (17%) in the experimental arm and 12 (24%) in the conventional arm died Secondary Outcomes: Remission not achieved in 6 (11%) in experimental arm, 7 (14%) in the conventional arm (*p* = 0.71) Relapse occurred in 20 (44%) in experimental arm, 41 (29%) in conventional arm	Part of the primary outcome of the study
Puechal et al., 2017, France	RCT	EGPA, PAN, MPA without features of poor prognosis	AZA (2 mg/kg/day) and GC (1 mg/kg/day for 3 weeks then tapered)	Placebo and GC (1 mg/kg/day for 3 weeks then tapered)	Combined rate of remission induction failures and minor or major relapses at month 24	95 patients (51 EGPA patients) Primary Outcome: 22 (48%) in the AZA group and 24 (49%) in the placebo group met end point (NS) Secondary Outcomes: No difference in initial relapse rates, relapses	Similar AE and SAE between groups
Wechsler et al., 2017, USA	RCT	Relapsing or refractory EGPA who received treatment for 4 weeks and on stable doses of GC	Mepolizumab 300 mg subcutaneous every 4 weeks plus standard care for 52 weeks (*n* = 68)	Placebo and standard care for 52 weeks (n = 68)	Accrued weeks of remission and proportion of participants in remission at weeks 36 and 48	136 patients Primary Outcomes: *Accrued weeks of remission*: 28% in Mepolizumab and 3% in placebo arm had > 24 weeks in accrued remission (significant) Proportion in remission at week 36 and 48: 32% in Mepolizumab and 3% in placebo group (significant) Secondary Outcomes: *Remission failure:* 47% failed in Mepolizumab group, 81% failed in placebo group	Similar AE between groups (Mepolizumab: 97%, Placebo: 94%). Serious AEs: Mepolizumab 18%, Placebo: 26%
Maintenance Trials							
Koike et al., 2015, Japan	RCT	EGPA patients with chronic residual peripheral neuropathy after disease remission	Group A: IVIG (0.4 g/kg for 5 days) followed by placebo then placebo (n = 8) Group B: Placebo, followed by IVIG (0.4 g/kg for 5 days), then placebo (*n* = 8) Group C: Placebo, followed by placebo, then IVIG (0.4 g/kg for 5 days) (*n* = 7) *Treatments provided at 2 week intervals*		Amount of change in the MMT sum score 2 weeks after IVIG administration	23 patients MMT change after IVIG: 7.13, significant increase when compared to baseline scores Scores were increased significantly after 4, 6, 8 weeks after observation	AE in 14 patients (61%). Headaches (*n* = 4) and elevated ALT (*n* = 3) were most common

*RCT* Randomized Controlled Trial, *PLEX* Plasma exchange, *SAE* Serious Adverse Event, *AE* Adverse Event, *GC* Glucocorticoids, *CYC* Cyclophosphamide, *NS* not significant, *AZA* Azathioprine, *MMT* Manual Muscle Testing, *ALT* Alanine Aminotransferase, *LFT* Liver function tests, *PAN* Polyarteritis Nodosa, *GPA* Granulomatosis with Polyangiitis, *MPA* Microscopic Polyangiitis

Based on a prospective cohort study, it is recommended that EGPA patients without life or organ threatening manifestations can be treated with corticosteroid monotherapy because of excellent 5-year survival rates, although relapse rates are high on a monotherapy regimen [42]. Puechal et al. performed a trial that evaluated the addition of AZA to corticosteroids for induction [43]. The study concluded no additional accrued benefits by adding AZA, with no differences in induction failures or relapses within 2 years (47% versus 49%) [41]. It is likely that a different adjunctive therapy is required for induction in non-severe EGPA patients.

Unique to EGPA is the potential role of interleukin-5 (IL-5) blockade. Wechsler et al. performed a RCT on the use of mepolizumab, an anti-IL-5 monoclonal antibody, in patients with relapsing or refractory EGPA [44]. Participants were either randomized to mepolizumab or to placebo. The two primary end points were the accrued weeks of remission at 52-weeks and the proportion of patients in remission at weeks 36 and 48. The mepolizumab group met the two primary end points with significantly more accrued weeks of remission and a higher percentage of patients in remission. However, 47% of participants did not achieve remission, and it is unclear why certain patients responded better than others. AEs were similar between groups, while serious AEs were slightly higher in the placebo group (26% versus 18%), although some of the events may be attributable to underlying EGPA activity.

RTX has not been studied by controlled trial in EGPA patients, although a few observational studies have reported successful use in both induction and maintenance [45, 46]. The adjunctive use of PLEX to CYC has been evaluated in an early study, which reported no additional benefit in survival [47].

## Pediatric considerations

Few studies have evaluated the outcomes of pediatric AAV (pAAV) or compared these with adult-onset AAV (aAAV). While some conflicting results exist, it appears that disease severity in pAAV is similar or slightly higher than in aAAV. Rottem et al. prospectively compared 23 pediatric GPA patients with 135 adult-onset patients and found that remission rates, relapse rates, and serious AE rates were similar [48]. Sacri et al. found that renal impairment occurred in 90% of pAAV at disease onset, which is more common than reports in aAAV which range from 10 to 20% at diagnosis to 60–80% during the disease course [49]. Iudici et al. compared 35 pAAV patients with 151 aAAV patients in a matched case-control study [50]. Both groups received similar induction therapy, most commonly corticosteroids and IV CYC. The authors report that by 5 years, pAAV patients had higher relapse rates, accumulated more damage, and were more likely to remain on corticosteroids and immunosuppressive agents than their adult counterparts. Eleven percent of pAAV and 9% of aAAV patients died.

To date, there have been no RCTs evaluating treatment regimens in pAAV. The four largest cohort studies assessing contemporary outcomes in pAAV found that the majority of patients received corticosteroids and CYC for remission induction, followed by AZA or MTX for remission maintenance (Table 5). The definitions and rates of remission and relapse varied between studies. Sacri et al. reported disease remission in 92% of pAAV patients, and a relapse rate of 41% [51]. In a single-centre study from Toronto [52], all 20 pAAV followed for a minimum of 6 months achieved remission; the relapse rate was 75% at a median follow-up of 10 months. Iudici et al. reported inactive disease in all pediatric EGPA and MPA patients and 68% of GPA patients at the last follow-up visit (median 96 months), however many of these patients continued to be on therapy, including many on corticosteroids [51].

**Table 5:** Pediatric Studies in AAV

Reference, Country	Study Design	Patient Selection	Induction therapy	Maintenance therapy	Therapy-related outcomes	Adverse Events
Akikusa et al., 2007, Canada	Retrospective 1984–2005	*n* = 25 GPA 100%	GC 100% CYC 76% AZA 40% MTX 32%	n = 7* AZA (n = 3) MTX (n = 4)	100% achieved remission (median 5 months, range 3–6 months) 75% relapse rate (median 10 months)	12 hospitalizations in 5 patients for infections 0 malignancies 0 deaths
Iudici et al., 2015, France	Retrospective	*n* = 35 GPA 71.4% EGPA 17.1% MPA 11.4%	GC 91.4% GC + IS 77.1% CYC IV 54.3% CYC PO 5.7% AZA 8.6% MTX 5.7% SZ 2.9% PLEX 2.9%	–	Inactive disease at last follow-up (*n* = 33, median 96 months): GPA 68.2% on treatment EGPA 100% on treatment (50% on GC + IS) MPA 100% on treatment (66.7% on GC + IS) Relapse rates: 76% overall (GPA 83%, EGPA 50%, MPA 33%)	9 infections 1 pancreatitis (GC) 2 cataracts (GC) 1 transaminitis (AZA) 1 hematologic (CYC) 1 hypertension (GC) 4 deaths
Sacri et al., 2015, France	Retrospective 1986–2011	*n* = 66 GPA 42% MPA 58%	GC 100% IV 86%, PO 14% CYC IV 47% CYC PO 20% RTX 13.6% MMF 4.5% PLEX 16.7%	GC alone 28.8% GC + AZA/ MTX/ MMF 63.6% AZA alone 1.5% None 6%	92.4% achieved remission Post-induction: 70% achieved remission 24.2% (*n* = 16) had refractory disease; 15/16 achieved secondary remission with addition of CYC (n = 8), IVIG (n = 2), RTX (n = 3) PLEX (n = 3) 40.9% relapse rate (median 29 mos)	4 deaths
ARChiVe, Morishita et al., 2017, International	2004–2007 retrospective, 2007–2008 prospective	*n* = 105 GPA 81% MPA 13% EGPA 6%	GC 100% (IV pulse 70%) CYC 70.5% MTX 16.2% RTX 13.3% AZA 1.9% MMF 1.0% PLEX 23.8%	AZA 42.3% MTX 22.8% MMF 13.3% CYC 9.5% RTX 9.5% None 4.7%	44/105 (42%) achieved remission by 12 months 21/44 (48%) discontinued GC 41/44 (93%) remained on maintenance 24% relapse rate	80 hospitalizations in 43 patients: 46% flares 16% infection 5% treatment-related 15% other disease-related 18% unrelated to vasculitis 0 deaths

*GPA* Granulomatosis with Polyangiitis, *EGPA* Eosinophilic granulomatosis with polyangiitis, *MPA* Microscopic polyangiitis, *GN* Glomerulonephritis, *GC* Glucocorticoids, *CYC* Cyclophosphamide, *AZA* Azathioprine, *MTX* Methotrexate, *RTX* Rituximab, *PLEX* Plasmapheresis, *IS* Immunosuppressant, *SZ* Sulfasalazine, *MMF* Mycophenolate mofetil, *IVIG* Intravenous immunoglobulin

*7 patients were treated with a remission induction/maintenance regimen

The largest pAAV cohort to date, the ARChiVe (A Registry for Childhood Vasculitis: e-entry) cohort [10], evaluated the outcomes of 105 pAAV patients and found that 42% achieved remission by 12 months (remission defined as PVAS of 0 on < 0.2 mg/kg/day corticosteroids or equivalent), and 61% had inactive disease on higher doses of corticosteroids. All but 3 patients in remission were on maintenance therapy at 12 months, and 48% had discontinued corticosteroids. Rates of remission were similar for those treated aggressively (CYC and/or RTX, 43%) versus moderately (MTX, AZA or MMF, 30%); those treated aggressively had higher baseline PVAS scores. Twenty-four percent of patients experienced minor relapses after achieving inactive disease. The remission rate of 42% in this study is lower than that reported in the adult studies, which ranges from 53 to 93% [14, 17, 22] but direct comparison is complicated by differences in study design, remission definitions, and corticosteroid regimens. Sixty-three percent of the ARChiVe cohort had evidence of damage by 12 months, compared to 87% reported in grouped adult data [52]. Given that we have speculated greater organ reserve in children compared to adults, this high rate of damage in children only 12 months after diagnosis is concerning and further suggests that children with AAV may have a more severe disease course than their adult counterparts.

The Single Hub and Access point for Pediatric Rheumatology in Europe (SHARE) initiative recently developed consensus-based guidelines for the management of rare pediatric vasculitides. These included recommendations for pAAV (including EGPA) along with Polyarteritis Nodosa and Takayasu Arteritis [53]. Given the paucity of pediatric-specific evidence and highly variable clinical practice amongst various centers, the aims of the recommendations were to define the minimum standard of care for these patients. A large portion of the guidelines is directed at establishing the diagnosis of systemic vasculitis in a pediatric patient.

Adult-derived literature was primarily used to address pAAV treatment recommendations, as the quality of pediatric evidence was poor and largely based on descriptive studies. Induction recommendations for severe disease largely remain similar to adult guidelines, which included corticosteroids and ivCYC as primary agents. The guidelines stress the importance of IV over PO CYC use given the lower cumulative toxicity but similar efficacy. Interestingly, PLEX was defined as a “typical” initial induction agent in severe AAV patients, while adult guidelines generally describe it as an adjunctive agent with insufficient evidence to support its use as a first-line therapy. Furthermore, while adult guidelines strongly support the use of RTX as a first-line remission induction therapy, the same recommendation was not made in the SHARE guidelines, and was considered a second or third line induction agent. Unfortunately, the guidelines do not distinguish severe from limited AAV, and do not provide any specific treatment recommendations for pediatric patients with limited disease, which could potentially lead to confusion or possible over-treatment of a unique subgroup of pAAV patients. Similar agents have been recommended for maintenance therapies.

## Future directions

There are questions that remain unanswered in AAV management. In the realm of induction management, LoVAS (Low-dose Glucocorticoids Plus Rituximab Versus High-dose Glucocorticoids Plus Rituximab for Remission Induction in ANCA-associated Vasculitis) is a trial currently underway to evaluate whether corticosteroid regimens can be used in lower doses when RTX is used as the induction agent [54]. The PEXIVAS study provides some preliminary suggestion that regimens utilizing lower doses of corticosteroids do not impact rates of severe outcomes (such as death or ESRD), but final results are pending [21]. The CLEAR study was a recent trial that demonstrated that Avacopan, a C5 receptor inhibitor, could potentially replace or reduce corticosteroid doses, bringing forward unique therapies with a possible steroid-sparing role [55].

With regards to maintenance, the MAINRITSAN3 is a RCT comparing RTX for 46 months compared to 18 months. The RITAZAREM trial is an ongoing study using higher dose RTX in patients with relapsing disease [56]. TAPIR (The Assessment of Prednisone In Remission Trial) is a trial comparing continuation of low-dose corticosteroid versus stopping corticosteroid entirely in GPA patients during maintenance.

Given that EGPA remains understudied in comparison to the other AAVs, further research is needed to determine the efficacy of conventional immunosuppressants and RTX in EGPA, and the optimal patient candidates and dosing regimen for mepolizumab. ANCA-negative patients, excluded by most trials to date, also remain understudied, constituting another future research priority.

Evidence-based guidelines for pAAV management remain sparse. The EULAR/EUVAS guidelines do not comment on the pediatric population. The CanVasc recommendations make 4 pediatric-specific statements, primarily suggesting that pediatric patients should be treated according to adult guidelines [35]. As previously mentioned, the SHARE guidelines for the management of pAAV have been developed in order to set the minimum standard of care when it comes to systemic vasculitis treatment [53]. The majority of treatment recommendations however, were based on low-quality pediatric evidence, expert opinion, or extrapolated from adult studies. While these recent guidelines address the pediatric rheumatology community’s desire and need for more pediatric specific recommendations [57], evaluating its uptake and usefulness is warranted. Additional multi-center studies in pAAV are required to address questions around efficacy and toxicity of existing therapies in the pediatric setting, so that pediatric guidelines may incorporate higher quality evidence.

## Conclusion

Significant progress has been made in our understanding of the management and outcomes of AAV over the last two decades. Future studies should be directed towards addressing the remaining unanswered questions, which include determining the optimal duration and regimen of AAV induction and maintenance therapy, improving our understanding of EGPA management, and developing evidence for pAAV to better inform pediatric-specific treatment guidelines.

## Data Availability

Not applicable. Published data that support the findings of this study are included in the article as part of the review.

## Abbreviations

aAAV: Adult-onset ANCA-Associated Vasculitis
AAV: ANCA-associated Vasculitis
AEs: Adverse Events
ANCA: Anti-neutrophil cytoplasmic antibody
AZA: Azathioprine
BVAS: Birmingham Vasculitis Activity Score
CanVasc: Canadian Vasculitis research network
CARRA: Childhood Arthritis & Rheumatology Research Alliance
CYC: Cyclophosphamide
EGPA: Eosinophilic Granulomatosis with Polyangiitis
ESRD: End stage Renal Disease
EULAR: European League Against Rheumatism
GPA: Granulomatosis with Polyangiitis
ivCYC: Intravenous Cyclophosphamide
MMF: Mycophenolate Mofetil
MPA: Microscopic Polyangiitis
MTX: Methotrexate
pAAV: Pediatric ANCA-Associated Vasculitis
PLEX: Plasma Exchange
poCYC: Oral Cyclophosphamide
poMTX: Oral Methotrexate
PRES: Pediatric Rheumatology European Society
PVAS: Pediatric Vasculitis Activity Score
RCT: Randomized Controlled Trial
RTX: Rituximab
SHARE: Single Hub and Access point for Pediatric Rheumatology in Europe
VDI: Vasculitis Damage Index
